# PP43 Budget Impact Analysis Of Next-Generation Sequencing Coverage In Taiwan’s National Health Insurance

**DOI:** 10.1017/S0266462324002150

**Published:** 2025-01-07

**Authors:** Hui-Ru Chang, Grace Li Ying Huang, Shou-Yu Lin, Shyr-Yi Lin

## Abstract

**Introduction:**

The exploration of molecular characteristics has emerged as a prominent trend to advance precision medicine. The utilization of genetic testing to guide therapy is integral to precision medicine. This study aims to investigate the potential patient populations for the reimbursement of next-generation sequencing (NGS) and assess the budget impact from the perspective of Taiwan’s single insurer, the National Health Insurance Administration.

**Methods:**

To comprehend the scope for medicines with companion diagnostics (CDx) involved, we analyze the U.S. Food and Drug Administration-approved/cleared diagnostic tests, conduct a literature review to identify medicines approved by the European Medicines Agency that require a CDx, and identify the medicines with CDx involved covered by the National Health Insurance (NHI) in Taiwan. Subsequently, we explore the potential reimbursement indications for NGS testing and conduct a budget impact analysis to evaluate the expected financial impact for the NHI over a five-year period. Furthermore, sensitivity analyses are conducted to deal with uncertainty.

**Results:**

We have compiled 13 cancer types for which NGS can serve as a companion diagnostic. These encompass non-small-cell lung cancer, colorectal cancer, breast cancer, ovarian cancer, biliary tract cancer, acute myeloid leukemia, acute lymphoblastic leukemia, melanoma, cholangiocarcinoma, prostate cancer, pancreatic cancer, gastrointestinal stromal tumor, and thyroid cancer/medullary thyroid cancer. The implementation of NGS reimbursement in NHI will benefit 25,000 to 30,000 patients undergoing targeted therapies. The projected incremental budget impact ranges from TWD570 million to TWD650 million (USD19 million to USD22 million) over five years.

**Conclusions:**

This study focuses on evaluating the financial impact of incorporating NGS testing into NHI reimbursement for relevant cancer drug indications. The findings can serve as references for the planning of reimbursement policies. However, with the advancement of precision medicine, it is foreseeable that there will be a broader range of applications for NGS, and its cost will gradually decrease.

